# Norepinephrine transport-mediated gene expression in noradrenergic neurogenesis

**DOI:** 10.1186/1471-2164-10-151

**Published:** 2009-04-08

**Authors:** Yao Fei Hu, Marc G Caron, Maya Sieber-Blum

**Affiliations:** 1 Department of Cell Biology, Neurobiology and Anatomy, Medical College of Wisconsin, Milwaukee, WI 53226, USA; 2 Department of Cell Biology and Medicine, Duke University, Durham, NC 27710, USA; 3 Institute of Human Genetics and North East England Stem Cell Institute, Newcastle University, Newcastle upon Tyne, NE1 3BZ, UK

## Abstract

**Background:**

We have identified a differential gene expression profile in neural crest stem cells that is due to deletion of the norepinephrine transporter (NET) gene. NET is the target of psychotropic substances, such as tricyclic antidepressants and the drug of abuse, cocaine. NET mutations have been implicated in depression, anxiety, orthostatic intolerance and attention deficit hyperactivity disorder (ADHD). NET function in adult noradrenergic neurons of the peripheral and central nervous systems is to internalize norepinephrine from the synaptic cleft. By contrast, during embryogenesis norepinephrine (NE) transport promotes differentiation of neural crest stem cells and locus ceruleus progenitors into noradrenergic neurons, whereas NET inhibitors block noradrenergic differentiation. While the structure of NET und the regulation of NET function are well described, little is known about downstream target genes of norepinephrine (NE) transport.

**Results:**

We have prepared gene expression profiles of in vitro differentiating wild type and norepinephrine transporter-deficient (NETKO) mouse neural crest cells using long serial analysis of gene expression (LongSAGE). Comparison analyses have identified a number of important differentially expressed genes, including genes relevant to neural crest formation, noradrenergic neuron differentiation and the phenotype of NETKO mice. Examples of differentially expressed genes that affect noradrenergic cell differentiation include genes in the bone morphogenetic protein (BMP) signaling pathway, the *Phox2b *binding partner *Tlx2*, the ubiquitin ligase *Praja2*, and the inhibitor of Notch signaling, *Numbl*. Differentially expressed genes that are likely to contribute to the NETKO phenotype include dopamine-β-hydroxylase (*Dbh*), tyrosine hydroxylase (*Th*), the peptide transmitter 'cocaine and amphetamine regulated transcript' (*Cart*), and the serotonin receptor subunit *Htr3a*. Real-time PCR confirmed differential expression of key genes not only in neural crest cells, but also in the adult superior cervical ganglion and locus ceruleus. In addition to known genes we have identified novel differentially expressed genes and thus provide a valuable database for future studies.

**Conclusion:**

Loss of NET function during embryonic development in the mouse deregulates signaling pathways that are critically involved in neural crest formation and noradrenergic cell differentiation. The data further suggest deregulation of signaling pathways in the development and/or function of the NET-deficient peripheral, central and enteric nervous systems.

## Background

The NET is a Na^+ ^and Cl^-^- dependent transporter, which is expressed by noradrenergic neurons. NET function in adult noradrenergic neurons is the clearing of secreted NE from the synaptic cleft via selective high-affinity uptake [1,2]. Drugs that block NE transport, such as the tricyclic antidepressant desipramine and the drug of abuse, cocaine, inhibit NE transport [1,2] and differentiation of cultured neural crest cells into noradrenergic neuroblasts [3,4]. NETKO mice have reduced body temperature (~1°C) and reduced body weight (~20%), they are supersensitive to psychostimulants, such as cocaine and amphetamine, they have reduced intracellular NE, increased NE synthesis and elevated extracellular NE [5]. In humans, abnormal NET function leads to orthostatic intolerance and is involved in depression, anxiety, attention deficit hyperactivity disorder (ADHD), and autonomic dysfunction [6-8]. NET may have additional functions during noradrenergic cell differentiation, as NET protein is expressed in a variety of different tissues in avian and mouse embryos [9]. NET expression in mouse embryonic neural crest cells is regulated by the autocrine growth factors, neurotrophin-3 (NT-3), fibroblast growth factor-2 (FGF-2) and transforming growth factor-β1 (TGF-β1; ref. [10]). The role of NET and the regulation of its function in noradrenergic homeostasis and NE signaling are well established. NET function is regulated by extracellular and intracellular signaling pathways that involve several associated proteins, including the SNARE protein syntaxin 1A, protein phosphatase 2A (PP2A) catalytic subunit (PP2A-C), PICK1, Hic-5, and PP2A anchoring subunit (PP2A-Ar) [11-13]. Little is known, however, on how absence of the NET gene affects differentiation of neural crest stem cells into noradrenergic cells.

Here we report results obtained with LongSAGE gene expression profiling and analyses on differentiating noradrenergic neurons/progenitors from the embryonic neural crest, the adult superior cervical ganglion and the locus ceruleus. SAGE has been developed by Velculescu et al [14] as a tool to quantify the transcriptome. It is based on the isolation and sequencing of unique sequences (tags) from defined positions at the 3' end of each mRNA molecule. SAGE has the advantage of high-efficient gene identification, which allows for unbiased and comprehensive analysis of a large number of differentially expressed genes without prior knowledge of the genes. The principle of LongSAGE is the same as in the original approach, except that it uses another type IIS restriction enzyme, *Mme1*, to generate 17 bp tags, rather than the 10 bp tags in conventional SAGE [15]. Thus LongSAGE allows for annotation of a larger portion of tags than SAGE.

## Results and discussion

### Time course of mouse NET gene expression and function during wild type neural crest cell differentiation in vitro

**Figure 1:**
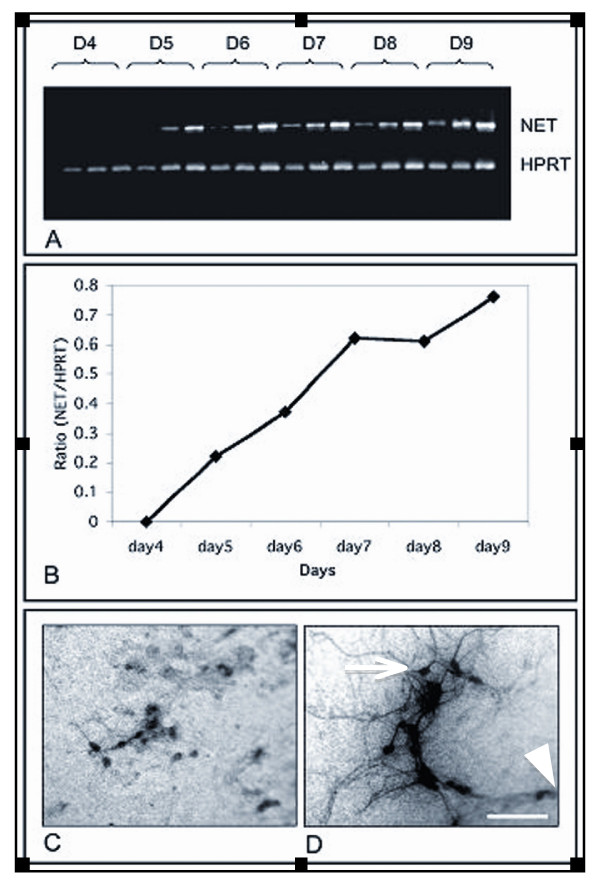
**Time course of expression of NET mRNA and NET function in cultured mouse neural crest cells**. (A) Semi-quantitative RT-PCR of NET mRNA on culture days 4 – 8 (upper) and Hprt (lower) at 28, 30 and 32 cycles each. Based on these results NET was subsequently amplified at 30 cycles. (B) Ratio *Net*/*Hprt *in triplicate during culture days 4 – 9 at 30 cycles of amplification. Data are expressed as average of three samples; error bars represent standard deviation. (C) Morphology of 3H-NE uptake-positive cells at day 3 of culture resembles immature neural crest cells. (D) At day 7 of culture, many 3H-NE uptake-positive cells have the morphology of mature sympathetic neuroblasts (arrow); NET uptake positive cells with undifferentiated morphology were still present (arrowhead). These observations indicated that at day 7, culture contain NE uptake-positive progenitors as well as neuroblasts with a functional NET. They are multipolar and extend long processes. Bar, (C, D) 100 μm.

To determine the optimal stage of in vitro development for RNA collection, we performed a time-course of NET expression and function in wild type mouse neural crest cell cultures. Both, NET mRNA (Fig. 1A, B) and high affinity ^3^H-norepinephrine uptake-positive cells (Fig. 1C) were first detected on culture day 5 in a subset of neural crest cells. At culture day 5, uptake-positive cells lacked processes and showed the morphology of undifferentiated neural crest cells (Fig. 1C). By culture day 7, many ^3^H-NE uptake-positive cells were multipolar with long processes and they tended to form aggregates (Fig. 1D arrow), whereas others showed a functional NET but had undifferentiated morphology as determined by the absence of processes (Fig 1D, arrowhead). Expression of catecholamine biosynthetic enzymes in mouse neural crest cell cultures starts around culture day 5 and newly catecholamine-positive cells continue to appear in progressively larger numbers, as stem cells persist for several more weeks in culture, self-renew and their progeny continue to differentiate. Thus day 7 cultures capture all stages of in vitro development. They contain neural crest stem cells, undefined NET-negative progenitor cells, cells with NET function and immature morphology, as well as cells with NET function and neuronal morphology as judged by the elaboration of long processes (Fig. 1D). For this reason day 7 cultures were chosen as a source of RNA for gene expression profiling. For the purpose of the present study we use expression of catecholamine biosynthetic enzymes and elaboration of processes as measures for neuronal differentiation. Among cells with morphology of differentiated cells, ^3^H-NE uptake-positive cells with neuronal morphology were observed only, indicating that in these cultures functional NET was limited to differentiating neuroblasts/neuronal progenitors. This notion is supported by the complete absence in the longSAGE libraries of differentially expressed genes that are characteristic for non-neuronal neural crest derivatives, such as smooth muscle cells, bone/cartilage cells or pigment cells.

### Summary of LongSAGE libraries and LongSAGE tag-to-gene mapping

We collected at total of 25,958 long-tags from NETKO neural crest RNA (Table 1). The library has been deposited in Gene Expression Omnibus (GEO) at  under GEO accession number GSE11788. The wild type library has been deposited previously and has been included by NCBI in the present series.

**Table 1:** LongSAGE Tags distribution in WT and NETKO neural crest cell LongSAGE libraries.

	WT		NETKO	
	
	Unique Tags	Total Tags	Unique Tags	Total Tags
Total	16,054	34,404	12,618	25,958
Count 1	11,993 (74.7%)	11,993 (34.9%)	9,494 (75.2%)	9,494 (36.6%)
Count 2–5	3,262 (20.3%)	8,772 (25.5%)	2,550 (20.2%)	6,916 (26.6%)
Count 6–19	632 (3.9%)	5,786 (16.8%)	449 (3.6%)	4,208 (16.2%)
Count ≥ 20	167 (1.1%)	7,853 (22.8%)	125 (1.0%)	5,340 (20.6%)

The wild type library consisted of 16,054 unique long-tags, whereas the NETKO library contained 12,618 unique LongSAGE tags (Table 1). These unique tags were matched to the LongSAGE database (NCBI; ) for gene identification. Only 167 (1.1%) LongSAGE tags in the wild type library and 125 (1.00%) LongSAGE tags in the NETKO library were present in more than 20 copies (Table 1). Ninety-five percent of LongSAGE tags in the wild type library and 95.4% LongSAGE tags in the NETKO library were represented by 5 or fewer copies (Table 1). This distribution is consistent with that observed in other cell types with conventional SAGE [16,17].

Of the unique LongSAGE tags, 10,536 (65.6%) tags in the wild type library and 8,657 (68.6%) tags in the NETKO library could be matched to known expressed sequences. 5,518 (34.4%) tags in the wild type library and 3,961 (31.4%) tags in the NETKO library were tags without matches to known sequences. They could represent novel genes or sequencing errors. Of the matched LongSAGE tags, 8,652 (82.1%) LongSAGE tags in the wild type library and 7,622 (88%) LongSAGE tags in the NETKO library were single-matched tags. Sequences that matched to more than one sequence located in different Unigene clusters, 1,884 (17.9%) LongSAGE tags in the wild type library and 1,035 (12%) in the NETKO library, were excluded from analysis.

### Quality and equality of the wild type and NETKO LongSAGE libraries

Several lines of evidence show the quality and equality of the two LongSAGE libraries. First, the tag distribution between the two libraries and the LongSAGE tag-to-gene mapping in both libraries were similar (Table 1). Second, as expected, most genes expressed by in vitro differentiating neural crest cells in day 7 cultures were unchanged because of the deletion of the NET gene (Table 1). As an additional quality control, we have analyzed the top 100 tags of both libraries (Additional files 1 and 2; top 50 tags shown). These tags accounted for 18.28% in the wild type library and 18.56% in the NETKO library. Eighty-five of the top 100 tags, and 42 of the top 50 tags, were common to both libraries (Additional files 1 and 2). Third, the expression of common house keeping genes, such as beta-actin, glyceraldehyde-3-phosphate dehydrogenase (GAPDH), hypoxanthine guanine phosphoribosyl transferase (HPRT), ribosomal protein L13, beta-2 microglobulin, and ubiquitin C were expressed at similar levels in both libraries (p > 0.1; Table 2).

**Table 2:** Abundance of house keeping genes in both libraries.

Description	Unigene	WT(TPM)	NETKO(TPM)	P value
actin, beta, cytoplasmic	Mm.133292	4234	4875	0.12
glyceraldehyde-3-phosphate dehydrogenase	Mm.5289	1131	1794	0.24
hypoxanthine guanine phosphoribosyl transferase	Mm.18675	232	273	0.48
ubiquitin C	Mm.331	406	507	0.27
beta-2 microglobulin	Mm.163	87	117	0.69
ribosomal protein L13	Mm.42578	812	975	0.38

Comparative analysis according to stringent criteria (≥ 1.5 fold difference and p < 0.05) identified 180 differentially expressed genes; 113 sequences were up-regulated in NETKO neural crest cells and 67 sequences were down-regulated (Additional files 3 and 4). Taken together, we provide a high quality NETKO LongSAGE gene expression library of medium size. By comparing it to an equivalent wild type library according to stringent criteria, we have identified a number of differentially expressed genes.

### Differential expression of noradrenergic biosynthetic enzymes

Deletion of the NET gene affects expression of noradrenergic biosynthetic enzymes [5] and NET function has been implicated in noradrenergic cell differentiation [3,4]. We validated by real-time PCR the differential expression of genes relevant to catecholamine metabolism in embryonic neural crest cells and in the adult locus ceruleus and superior cervical ganglion (Table 3). Dopamine-β-hydroxylase (*Dbh*) and tyrosine hydroxylase (*Th*), were significantly up-regulated in NETKO tissue in all three locations (Table 3), confirming equivalent data by Xu et al. [5]. Monoamine oxidase-A (MAO-A) was down regulated in all three tissues, whereas catechol O-methyltransferase (COMT) was not significantly affected (Table 3).

**Table 3:** Confirmation by quantitative PCR of differential expression of noradrenergic neuron-relevant genes.

**Genes**	**Unigene Number**	**LongSAGE**		**Quantitative PCR (ratio KO/WT)**		
		
		**WT**	**KO**	**Day 7 NCC**	**Adult LC**	**Adult SCG**
TH	Mm.1292	2	9	4.2 ± 0.4 (p = 0.001)	2.4 ± 0.2 (p = 0.04)	4.4 ± 0.2 (p = 0.03)
DBH	Mm.167781	4	14	4.1 ± 0.5 (p = 0.004)	3.1 ± 0.2 (p = 0.000)	6.8 ± 0.8 (p = 0.001)
MAO-A	Mm.21108	2	0	0.6 ± 0.0 (p = 0.006)	0.5 ± 0.0 (p = 0.002)	0.9 ± 0.1 (p = 0.04)
COMT	Mm.100940	0	1	1.1 ± 0.0 (p = 0.1)	1.7 ± 0.5 (p = 0.2)	1.4 ± 0.1 (p = 0.05)
Htr3a	Mm.4831	1	11	2.6 ± 0.6 (p = 0.004)	3.5 ± 0.3 (p < 0.0001)	4.0 ± 0.3 (p = 0.001)
CART	Mm.75498	12	22	2.0 ± 0.2 (p = 0.004)	3.9 ± 0.2 (p < 0.0001)	4.7 ± 0.3 (p = 0.001)
NET	Mm.57040	1	0	Not detected in KO	Not detected in KO	Not detected in KO
Pja2	Mm.41711	1	12	7.0 ± 1.8 (p = 0.04)	N/A	N/A
Numb1	Mm.458153	0	7	3.5 ± 0.5 (p = 0.006)	N/A	N/A
Hdac2	Mm.19806	12	1	-6.97 ± 1.8 (p = 0.021)	N/A	N/A
Tlx2	Mm.37	0	9	7.63 ± 1.2 (p = 0.04)	N/A	N/A

For determining mRNA levels, the 2^-ΔΔC^_T _method was used as described. For a given target, ΔC_T _was computed by subtracting C_T _for HPRT from each primer pair. C_T _and ΔΔC were computed by subtracting ΔC_T _for WT from ΔC_T _for NETKO. The difference of the expression level for each gene was expressed as 2^-ΔΔC^_T_. Statistical analysis was performed using the Student's *t *test. qPCR data are presented as mean ± S.E.M., and significant differences between KO and WT reported at the p < 0.05 level; n = 3.

**Figure 2:**
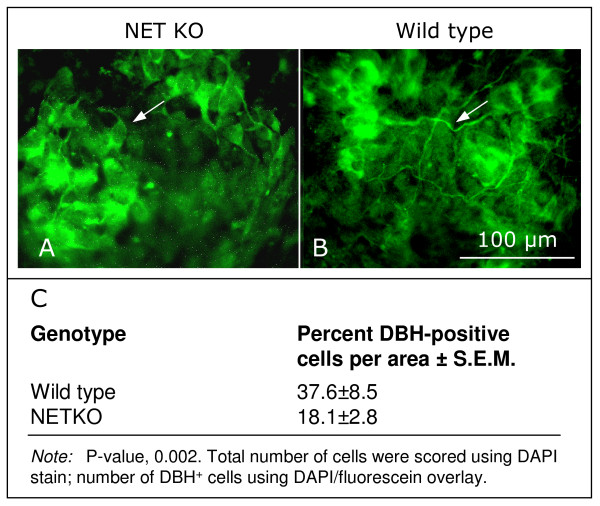
**Altered morphology and reduced numbers of DBH-immunoreactive cells in day 7 NETKO neural crest cultures**. (A) NETKO neural crest cells have no or short processes only (e.g., arrow). (B) By contrast, wild type DBH-positive cells have long processes (e.g., arrow). (C) Quantification of the number of DBH-immunoreactive cells expressed in day 7 wild type and NETKO neural crest cultures. Approximately half the number of DBH-positive cells is expressed in NETKO cultures compared to wild type cultures. Bar, (A, B) 100 μm.

The morphology and percentage of NETKO and wild type in vitro differentiating noradrenergic neural crest-derived cells differed (Fig. 2). In wild type neural crest cultures, many cells were DBH-immunoreactive and showed long processes (Fig. 2B), whereas DBH-immunoreactive NETKO cells had no processes or short extensions only (Fig. 2A). Furthermore, NETKO neural crest cultures contained only about half the number of DBH-immunoreactive cells per area compared to wild type cultures (Fig. 2C). The reduced numbers of in vitro differentiating NETKO neural crest-derived noradrenergic cells is in agreement with our earlier observations that NE uptake by the NET promoted noradrenergic differentiation, whereas NET blockers were inhibitory [3,4]. Taken together the data suggest that fewer neural crest cells differentiated into noradrenergic cells in the absence of NET, but that the cells that did become noradrenergic expressed higher transcript levels of biosynthetic enzymes that lead to the production of norepinephrine.

### Differential expression of Cart, Htr3a and Tlx2

Cocaine and amphetamine regulated transcript (*Cart*; currently *Cartpt*, Mm.75498), the serotonin receptor subunit, *Htr3a *(Mm.4831) and the T-cell leukemia homeobox 2 (*Tlx2*; Mm.37) were significantly more abundant in the NETKO LongSAGE library compared to the wild type library (Tables 3 and Additional file 3). Altered expression of all three genes may contribute to the NETKO phenotype.

**Figure 3:**
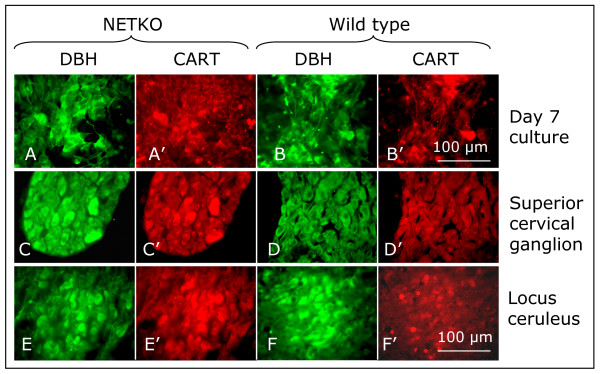
**Co-localization of CART and DBH immunoreactivities in wild type and NETKO neural crest cells, superior cervical ganglion and locus ceruleus**. (A, A', B, B'), NETKO and wild type neural crest cultures, respectively, at day 7. (C, C', D, D'), superior cervical ganglion of 10 week-old mice; (E, E', F, F'), Locus ceruleus from 10-week old wild type and NETKO mice. Wild type cells express CART at low levels. DBH and CART immunoreactivities co-localize in all three tissues. CART and DBH immunoreactivity appear somewhat brighter in NETKO tissue than in wild type tissues, suggesting increase in expression not only at the RNA but also at the protein level. It needs to be noted that immunofluorescence was not quantified. Bar, (A – F') 100 μm.

One aspect of the NETKO phenotype is hyper-responsiveness to psychostimulants, such as cocaine or amphetamine [5]. CART is a putative neurotransmitter, or co-transmitter, in the brain, in the adrenal gland and in neural crest-derived enteric ganglia [18-20]. *Cart *expression is up-regulated in response to acute administration of psychostimulants [21]. CART peptide co-localizes with noradrenergic neurons in the locus ceruleus, in noradrenergic C1 neurons and in the nodose ganglion [19-22]. The CART peptide modulates the activity of the striatal noradrenergic and the corticostriatal and hypothalamic serotonergic systems in the rat brain and it is involved in feeding, emotional and locomotor behavior [21]. It can produce anxiety-like effects in rodents [23]. We confirmed differential expression and co-localization of *Cart *by real-time PCR and at the protein level by immunocytochemistry in embryonic neural crest cultures, in the adult superior cervical ganglion and in the adult locus ceruleus (Table 3; Fig. 3). It is conceivable that elevated Cart expression in NETKO mice causes their hyper-responsiveness to psychostimulants. While there also seemed to be an increase in intensity of immunofluorescence for DBH and CART in NETKO tissue, we did not pursue quantification of fluorescence.

The A subunit of the 5-HT3 receptor, *Htr3a*, is 11-fold more abundant in the NETKO library than in the wild type library (Table 3; Additional file 3). We confirmed by real-time PCR this up-regulation of *Htr3a *in NETKO neural crest, locus ceruleus and superior cervical ganglion cells (Table 3). Serotonin (5-hydroxytryptamine) is a neurotransmitter that interacts with multiple receptors to mediate a wide range of effects, including involvement in anxiety and depression [24]. Additionally, *Htr3a *mRNA is present both in submucosal and myenteric ganglia in enterochromaffin cells of the gut, which activate the 5-HT3A receptor in extrinsic primary afferent neurons [25,26]. The 5-HT3A receptor is thus a link between gut and brain. Since 5-HT3A antagonists cause constipation, the function of the 5-HT3A receptor is considered important for normal enteric motility [27-29]. *Htr3a *over-expression therefore could affect serotonin signaling and thus peristalsis. In our present study, *Nsg2 *(neuron specific gene family member 2; Mm.3304) and *Cart *were significantly more abundant in the NETKO library than in the wild type library (Table 3; Additional file 3). Interestingly, both genes were found to be down-regulated in the Ret-deficient enteric nervous system [30]. Together, the two studies suggest that *Net *and *Ret *are upstream of *Nsg2 *and *Cart*, and that they have opposing effects on *Nsg2 *and *Cart *expression. This is of interest, as Ret-deficient mice have Hirschsprung's syndrome, i.e. absence of distal enteric ganglia.

T-cell leukemia homeobox 2 (*Tlx2*; aliases, *Enx, Hox11L.1, Hox11l1, NCX, Ncx1, Tlx1l1*, and *Tlx1l2*) was 12-fold more abundant in the NETKO LongSAGE library than in the wild type library (Table 3; Additional file 3). *Tlx2 *encodes a transcription factor downstream of BMP signaling, which is essential for the development of sympathetic neurons, as it is activated by, and binds to, Phox2B [31]. An imbalance between Tlx2 and Phox2b expression may affect autonomic nervous system development, as Phox2b is essential for the differentiation of neural crest cells into autonomic neurons [32].

In addition to *Htr3a, Cart *and *Tlx2 *are also expressed in the gastrointestinal tract. Cart is expressed in enteric neurons and is thought to serve a modulatory function in the enteric nervous system [33]. *Tlx2 *knock-out mice demonstrate lethal intestinal pseudo-obstruction and colonic hyperganglionosis, similar to human intestinal neuronal dysplasia [34-36]. Since *Tlx2 *is not only expressed in neural crest-derived enteric neurons, but also in visceral smooth muscle cells [37], perturbation of gastrointestinal function may not be limited to enteric nervous system dysfunction, but also be due to defects in the enteric smooth musculature.

Taken together, over-expression of *Cart, Htr3a and/or Tlx2*, is likely to cause a perturbation in noradrenergic cell differentiation and in enteric nervous system function in NETKO mice. Differential expression of the alpha(2A)-noradrenergic receptor, alpha(2C)-noradrenergic receptor [38] and neurotrophin-3 [39] were observed by Bönisch and collaborators by real-time PCR and at the protein level. We did not capture these genes in our libraries. The discrepancy could be due to differences in the starting material used, as we have collected RNA from embryonic neural crest stem cells, whereas in the other two studies [38,39] adult brain tissue was used. Conversely, these transcripts may have escaped detection in our libraries. The latter possibility is unlikely, as we have not observed the three genes in any of our 4 neural crest longSAGE libraries (ref [40] and this study).

### Differential expression of members of the Notch pathway in NETKO neural crest cells

Numbl (Numb-like) and APP (beta amyloid precursor protein) repress Notch activity [41]. Both *Numbl *and *App *were significantly increased in the NETKO library (Table 3; Additional file 3), suggesting decreased Notch signaling in NETKO cells. The Notch pathway is, however, essential for induction of the neural crest. It is required for initiation of BMP-4 expression, and thus neural crest identity, in cells at the boundary between somatic and neural ectoderm [42]. Notch signaling is involved in neural crest formation and noradrenergic cell differentiation, as well as in many other systems. Loss of Numbl function leads to a premature depletion of neuronal progenitor cells [43,44]. Since *Numbl *transcripts are significantly more abundant in NETKO neural crest cells (Table 3; Additional file 3), this result suggests that deletion of the NET gene causes noradrenergic precursor cells, such as neural crest cells, to preferentially remain in the neuronal progenitor cell compartment. The notion of decreased differentiation due to persisting progenitor state is supported by the 12-fold decrease in the expression of histone deacetylase 2 (*Hdac2*; Tables 3; Additional file 4). Overall, our data indicate perturbations in the Notch signaling pathway in NETKO cells, which is likely to affect neural crest formation and subsequent noradrenergic cell differentiation.

### Deregulation of the TGF-β and BMP signaling pathways

TGF-β signaling inhibits proliferation of neural crest cells and promotes their differentiation into autonomic neurons [45,46]. Praja2 (Pja2; neurodegeneration associated protein 1; Mm.41711), a RING H-2 protein with E2-dependent E3 ubiquitin ligase activity, is 12-fold more abundant in the NETKO library (Additional file 3) and 7-fold increased according to qPCR (Table 3). Praja2 ubiquitinates the Smad adaptor protein, Elf, which subsequently leads to its degradation and a decrease in Smad4 expression [47]. Smad4 is a critical member of TGF-β signaling, as it forms a complex with a receptor-regulated Smad (Samd1, Smad2). The complex subsequently serves as a transcripton factor for Tgf-β target genes [48]. In summary, elevated Pja2 expression can inhibit TGF-β signaling and therefore is likely to maintain neural crest cells in their progenitor state by blocking their differentiation into noradrenergic cells. Yet another important family of growth factors involved in noradrenergic differentiation are bone morphogenetic proteins (BMPs). BMP4 was found to be required for noradrenergic differentiation in chick embryos [49]. In agreement with this notion we found in the present study that BMP6 is significantly down-regulated in NETKO neural crest cells (Additional file 4).

## Conclusion

NET is an important gene in the central, autonomic and enteric nervous systems, as mutations in the NET gene have been shown to have profound influences in homeostasis and cognition. In this study we have primarily focused on the role of NET in embryonic neural crest development, but also have validated selected data in adult brain and peripheral nervous system tissues. Specifically, we have provided evidence that high-affinity uptake of norepinephrine through NET affects expression of genes that are involved in neural crest formation and in noradrenergic differentiation as measured by expression of catcholamine biosynthetic enzymes and elaboration of processes. The data further suggest changes in enteric nervous system function and possibly brain development/function in the absence of NET function. We have confirmed that expression of noradrenergic biosynthetic enzymes is altered in NETKO mice. Other pertinent differentially expressed genes addressed in detail in this work include Htr3a, *Numbl, App, Praja2 and Tlx2*. We have identified differentially expressed genes that are likely to contribute to the NETKO phenotype, that is over-expression in NETKO cells of *Th, Dbh, Cart, Htr3a *and *Tlx2*. Interestingly these genes are not only expressed in the neural crest-derived autonomic nervous system and in the brain, but they also have key functions in another neural crest derivative, the enteric nervous system. Other differentially expressed transcripts, as for instance Tgfb2 and Hoxa10 (Additional file 3), as well as Cdc51 and Hoxb9 (Additional file 4), play important roles in cell proliferation and differentiation but have not been addressed in the current study. Overall, we provide a valuable database for future investigations into NET function.

## Methods

### Genotyping of embryos and neural crest cell primary cultures

The animals were maintained in the transgenic mouse facility at the Medical College of Wisconsin, and all experiments were conducted in accordance with the "Guidelines for the Care and Use of Animals" approved by the Medical College of Wisconsin. Embryos were obtained from timed-pregnant females. The day vaginal plugs were observed was defined as day 0.5 of gestation. Genotyping was performed by PCR as described by Xu et al [5]. The NETKO strain has been back-crossed to the C57BL/6J background. Thus, C57BL/6J mice from the Jackson Laboratory were used for wild type cultures.

Neural crest cell primary cultures were prepared from embryos at day 9.5 of gestation, as we have described previously [9,40,45,50,51]. The dorsal trunk region posterior to the hind limb buds was dissected and treated with 1% trypsin (Difco, 1:250; Becton Dickinson, Sparks, MD). The neural tube was separated from other tissues of the trunk by gentle trituration. Forty-eight hours post-explantation, the neural tubes were carefully detached from the dishes and discarded. The neural crest cells, which remained on the collagen substratum were incubated at 37°C in a humidified atmosphere of 5% CO_2 _and 10% O_2 _[12]. The culture medium consisted of 75% alpha-MEM, 10% fetal bovine serum and 5% day 11 chicken embryo extract, and it was supplemented with 2.5 ng/ml basic fibroblast growth factor (FGF-2, Upstate Biotechnology, Lake Placid, NY), 10 ng/ml neurotrophin-3 (NT-3, Promega, Madison, WI), 100 ng/ml mouse stem cell factor (R&D System, Minneapolis, MN), and 10 nM arterenol (Sigma, St. Louis, MO) at. The culture medium and supplements was exchanged daily.

### High-affinity norepinephrine uptake

Neural crest cells with a functional norepinephrine transporter were identified *in situ *exactly as we have described previously [3,4]. Briefly, the cultures were rinsed with Hanks' balanced salt solution (HBSS) containing 1% bovine serum albumin (BSA). They were then incubated for 2 hours at 37°C with 0.5 ml of 0.5 μM [^3^H]-norepinephrine (40.8 Ci/mmol, Amersham Biosciences, Piscataway, NJ) in HBSS that also contained 1 mM ascorbic acid (Sigma, St. Louis, MO) and 0.1 mM of the monoamine oxidase inhibitor, pargyline (Sigma, St. Louis, MO), Subsequently, uptake of radioactive NE was terminated by rinsing the cultures 3 times with HBSS that contained 24 mM non-radioactive norepinephrine (d, l-arterenol; Sigma, St. Louis, MO), fixed with 4% paraformaldehyde in calcium-magnesium-free PBS for 20 min at room temperature, and rinsed again. The cultures were dried in a stream of cold air, coated in the dark with NTB2 emulsion (Kodak, Rochester, NY) for 2.5 min, and air dried in the dark. After 10 days of exposure at 4°C, autoradiographs were developed with D-19 (Kodak, Rochester, NY) and fixed with Rapid Fix (Kodak; Rochester, NY), mounted with mineral oil and a coverslip. They were then observed with a light microscope. Under these conditions, the NET inhibitor, desipramine, blocks uptake [3,4].

### LongSAGE library construction and data analysis

The RNA of 60 wild type and 60 NETKO neural crest cell cultures was isolated at culture day 7. Total RNA was isolated using TRIzol reagent (Invitrogen, Carlsbad, CA) according to the manufacturer's protocol. To avoid potential contamination with genomic DNA, total RNA was treated with DNase (Invitrogen, Carlsbad, CA). The LongSAGE libraries were constructed using the I-SAGElong kit (Invitrogen, Carlsbad, CA) according to manufacture's instructions. In brief, mRNAs were bound to Dynal oligo(dT) magnetic beads of the cDNA synthesis module, Invitrogen, Carlsbad, CA), mRNA transcripts were converted to cDNAs with biotinylated oligo(dT)18 as the primer (cDNA synthesis module, Invitrogen, Carlsbad, CA). The cDNA were digested with Nla III, and the 3' ends were recovered and bound to LS-adapter 1 and 2. Subsequently, the restriction enzyme, *MmeI*, was used to release the tags, which were ligated to form ditags. Ditags were amplified by PCR, the amplified ditags were isolated by using 12% polyacrylamide gel electrophoresis (PAGE) and digested again with Nla III to release the 34 bp LongSAGE ditags, which were purified by 12% PAGE. The ditags were concatemerized at their *Nla III *overhangs. Concatemers with minimum size of 500 bp were obtained by gel electrophoresis purification. They were ligated into the cloning vector pZEro-1 plasmid and transformed into TOP10 bacteria by electroporation. High-throughput sequencing was performed by Agencourt Bioscience Corporation (Beverly, MA). Both the 17 bp LongSAGE and the corresponding 10 bp SAGE tags were provided by Agencourt. Additional information about SAGE and LongSAGE technique can be found at .

LongSAGE data were analyzed with the SAGE2000 v 4.5 software (Invitrogen). Tags corresponding to linker sequences were discarded, and duplicate dimers were counted once only. Both 17 bp LongSAGE tags and corresponding 10 bp SAGE tags were extracted for further analysis. All tags were mapped to their corresponding genes using SAGEmap data from the National Center for Biotechnology Information (NCBI; ). After tag-to-gene mapping, putative function was annotated using the gene ontology (GO) database . All genes were annotated according to biological process. The libraries were normalized using the SAGE2000 software. Comparisons between the two LongSAGE libraries was carried out using statistical functions available in the SAGE2000 software for p-value calculation and Monte Carlo simulations. Tags with multiple matches were excluded, and tags that matched to the same Unigene cluster were combined. A p-value of <0.05 was considered significant.

### Real-time RT-PCR and semi-quantitative RT-PCR

For RT-PCR, mouse neural crest cell primary cultures and dissected tissue from adult mice (superior cervical ganglion and pontine brain stem containing the locus ceruleus) were dissolved with TRIzol reagent (Invitrogen, Carlsbad, CA). Total RNA was treated with DNase I (Invitrogen, Carlsbad, CA) to remove any traces of genomic DNA. First-strand cDNA was synthesized using the SuperScript III First-strand synthesis system for RT-PCR (Invitrogen, Carlsbad, CA), and primed by using oligo(dT) according to manufacturer's instructions.

The time course of NET gene expression in cultured neural crest cells was determined by semi-quantitative RT-PCR. Aliquots of the PCR products were resolved on 2% agarose gel containing ethidium bromide, and bands were visualized under UV illumination. The signal intensity was analyzed by computerized densitometry using the Molecular Dynamics STORM scanning system (Amersham Biosciences) as a ratio of a target gene over Hypoxanthine guanine phosphoribosyl transferase (*Hprt*).

Real-time PCR was performed in an Icycler (Bio-Rad, Hercules, CA) using Platinum SYBR green qPCR SuperMix UDG (Invitrogen, Carlsbad, CA), according to manufacturer's instructions. For each PCR product, a single narrow peak was obtained by melting curve analysis at the specific melting temperature, and a single band of the predicted size was observed by agarose gel electrophoresis. HPRT, which was expressed at nearly identical levels in both libraries, was used for normalization. For determining mRNA levels, the 2^-ΔΔC^_T _method was used as described [52]. The amplification efficiency of each target was evaluated from the cycle threshold (C_T_, the number of cycles to reach threshold) numbers obtained for serial cDNA dilution. For a given target, ΔC_T _was computed by subtracting C_T _for HPRT from each primer pair C_T_, ΔΔC_T _was computed by subtracting ΔC_T _for WT from ΔC_T _for NETKO. The difference of expression level for each gene expressed as 2^-ΔΔC^_T_. Standard curves were plotted for all primer sets with serial tenfold dilution of the cDNA samples. Overall efficiencies (*E*) of PCR were calculated from the slopes of the standard curves according to the equation: *E *= 10^(-1/slope)^. Target and reference genes showed similar efficiencies (>92%). Statistical analysis was performed with Student's *t *test, Data are presented as means ± S.E.M., and significant differences reported at the p < 0.05 level. Primer sequences are listed in Additional file 5.

### Immunocytochemistry

For immunocytochemistry, small tissue pieces and cell cultures were fixed with 4% paraformaldehyde in the cold overnight or for 30 minutes, respectively. Ten μm cryosections, or culture plates, were rinsed in phosphate buffered saline (PBS; 3 × 20 min), incubated with 4% normal goat serum (NGS) in PBS for 30 min, and subsequently incubated overnight at 4°C with rabbit anti-DBH antibody (Protos Biotech, New York) and chicken anti-CART antibody (Chemicon, Temecula, CA) respectively in PBS that contained 1% NGS and 0.1% Triton-X100 (Sigma). The secondary antibody (fluorescein-conjugated goat anti-rabbit IgG, and donkey anti-chicken IgG; Jackson ImmunoResearch Laboratory, PA) was diluted in 4% normal goat serum in PBS, added to the cultures and incubated in the dark for 1 hour at room temperature. Nuclei were stained with DAPI. The images were taken under the same exposure settings.

## Abbreviations

**APP**: beta amyloid precursor protein; **DBH**: dopamine-β-hydroxylase; **Cart/Cartpt**: catecholamine and amphetamine regulated transcript; **FGF-2**: fibroblast growth factor-2; **Hdac2**: histone deacetlylase 2; **Htr3a**: serotonin receptor subunit 3a; **LC**: locus ceruleus; **longSAGE**: long serial analysis of gee expression; **NCSC**: neural crest stem cell; **NE**: norepinephrine; **NT-3**: neurotrophin-3; **NET**: norepinephrine transporter; **Numbl**: Numb-like; **Pja2**: Praja2; **SCG**: superior cervical ganglion; **TH**: tyrosine hydroxylase; **TGF-βTgfb2**: transforming growth factor-beta; **Tlx2**: T-cell leukaemia homeobox2.

## Authors' contributions

MSB conceived and designed the project and wrote the paper. YFH participated in the design of the study, constructed the LongSAGE libraries, annotated the libraries, performed statistical analysis and contributed to the writing of the manuscript. CMG prepared and provided the NETKO mouse line. All authors read and approved the manuscript.

## Supplementary Material

Additional file 1 **Top 50 LongSAGE tags in the wild type library**. This table lists the 50 most abundant tags in the wild type library. Click here for file

Additional file 2 **Top 50 LongSAGE tags in the NETKO library**. This table lists the 50 most abundant tags in the NETKO library. Click here for file

Additional file 3 **Upregulated transcripts in NETKO library**. This file contains differentially expressed transcripts that are up-regulated in NETKO cells. Click here for file

Additional file 4 **Downregulated transcripts in NETKO library**. This file contains differentially expressed transcripts that are down-regulated in NETKO cells. Click here for file

Additional file 5 **List of primers used**. These two tables list the primers used. Click here for file
