# Supplementary material for: Norepinephrine transport-mediated gene expression in noradrenergic neurogenesis
Source: BMC Genomics. 2009 Apr 8;10:151. doi: 10.1186/1471-2164-10-151 (PMC2679758; doi:10.1186/1471-2164-10-151)
Supplement: Additional file 1 — Top 50 LongSAGE tags in the wild type library. This table lists the 50 most abundant tags in the wild type library. [file 1471-2164-10-151-S1.doc]

**ADDITIONAL FILE 1**: Top 50 LongSAGE tags in the wild type library

| WT top 50 Long Tag | WT | KO | unigene. | description |
| --- | --- | --- | --- | --- |
| AGCAGTCCCCTCCCTAG | 211 | 126 |  | No match |
| GCTGCCCTCCACCATAT | 183 | 198 |  | No match |
| ATACTGACATTTTGTAG | 183 | 163 | 328444 | Mus musculus transcribed sequence with weak similarity to protein prf:810024E (H.sapiens) 810024E cytochrome oxidase III [Homo sapiens] |
| GTGGCTCACAACCATCC | 155 | 114 | 2913 | multiple match |
| ATAATACATAAAAAAAA | 150 | 21 |  | No match |
| GGCAAGCCCCAGCGTCT | 138 | 118 | 297514 | Ribosomal protein L10A |
| GAAGCAGGACCAGTAAG | 134 | 87 | 4024 | cofilin 1, non-muscle |
| AGGCAGACAGTTGCTGT | 122 | 118 | 298110 | eukaryotic translation elongation factor 1 alpha 1 |
| GGCTTCGGTCTTTTTGA | 121 | 74 | 3158 | Ribosomal protein, large, P1 |
| TGGCTCGGTCACTTGGG | 117 | 82 | 298070 | actin, gamma, cytoplasmic |
| CCCTACTTCATCCTTTG | 115 | 66 | 14802 | H19 fetal liver mRNA |
| GCCTAATGTACACAAAG | 114 | 76 | 29105 | multiple match |
| GTGGCTCACAACCATCT | 110 | 98 | 38241 | multiple match |
| CCAGAACAGACTGGTGA | 105 | 66 | 259224 | Ribosomal protein L30 |
| GGATTTGGCTTGTTTGA | 100 | 83 | 318281 | Ribosomal protein, large P2 |
| ATGTCTCAAAGTAAAAG | 97 | 63 | 231463 | tubulin, alpha 2 |
| TGGGTTGTCTAAAAATA | 92 | 52 | 254 | tumor protein, translationally-controlled 1 |
| GGGGAAATCGCCAGCTT | 82 | 71 | 3532 | thymosin, beta 10 |
| GCAGGCACTCAATAAAT | 82 | 68 | 273538 | multiple match |
| TGTAGTGTAATAAAGGT | 81 | 63 | 260904 | Ribosomal protein S8 |
| GCCCGGGAATAAATTCA | 80 | 62 | 276337 | Ribosomal protein L17 |
| CAAGGTGACAGGCCGCT | 79 | 59 | 1129 | Ribosomal protein S2 |
| CCCTGAGTCCACCCCGG | 78 | 43 | 133292 | multiple match |
| TTGGTGAAGGAAAAAGC | 77 | 55 | 142729 | thymosin, beta 4, X chromosome |
| TATGTCAAGCTGGTGGA | 75 | 48 | 296178 | Ribosomal protein S12 |
| GGGAAGGCGGCAGCTCT | 74 | 54 | 296850 | Ribosomal protein S3a |
| TGACCCCGGGACCAAAT | 73 | 52 | 43005 | ubiquitin A-52 residue ribosomal protein fusion product 1 |
| GGAAGCCACTTTGACAG | 72 | 68 | 180003 | Ribosomal protein S27a |
| GCGGCGGATGGAGACTT | 68 | 61 | 43831 | lectin, galactose binding, soluble 1 |
| ATGACTGATAGCAAGTC | 68 | 47 | 29924 | ADP-ribosylation factor-like 6 interacting protein 1 |
| GAGCGTTTTGGGTCCAG | 68 | 37 | 5246 | peptidylprolyl isomerase A |
| TATCCCACGCCTGCTGG | 65 | 52 | 181721 | multiple match |
| CAGGCCACACAAGAGCC | 65 | 39 | 238973 | multiple match |
| CTAGTCTTTGTACACAA | 63 | 31 | 331739 | Ribosomal protein S29 |
| CTGTAGGTGATATTCCT | 62 | 41 | 301301 | Ribosomal protein S23 |
| TTCATTATAATCTCAAA | 62 | 22 | 19187 | prothymosin alpha |
| CTAATAAAGCCACTGTG | 61 | 69 | 298117 | Finkel-Biskis-Reilly murine sarcoma virus (FBR-MuSV) ubiquitously expressed (fox derived) |
| AGGAAGGCGGCGGCCAA | 61 | 33 | 11376 | Ribosomal protein L36 |
| GCCAAGTGGAGTTCCCC | 58 | 45 | 289431 | eukaryotic translation elongation factor 2 |
| GTTGCTGAGAAGCGGCT | 57 | 49 | 311053 | Ribosomal protein 10 |
| CGCCGCCGGCTCACCAA | 57 | 41 | 16423 | Ribosomal protein L35 |
| TAAAGAGGCCGTTTTGT | 55 | 55 | 372 | Ribosomal protein S26 |
| AACAGGTTCAATCAGCT | 55 | 43 | 292027 | Ribosomal protein S25 |
| CATTTTCTGGCAAAATC | 55 | 34 | 3862 | insulin-like growth factor 2 |
| CCCTGGGTTCTGCCCGC | 54 | 39 | 30357 | ferritin light chain 1 |
| GGCAGCTCCCACCTATC | 54 | 24 | 283283 | Transgelin |
| CGCTGGTTCCAGCAGAA | 53 | 43 | 250335 | RIKEN cDNA 2010203J19 gene |
| GTCTGCTGATGGCCAGA | 51 | 46 | 5305 | guanine nucleotide binding protein, beta 2, related sequence 1 |
| GATGACACCAGCCGCTC | 51 | 37 | 200920 | Ribosomal protein S28 |
| CAAAAATAAAAGCCGCA | 49 | 95 | 70666 | enolase 1, alpha non-neuron |
