# Supplementary material for: Norepinephrine transport-mediated gene expression in noradrenergic neurogenesis
Source: BMC Genomics. 2009 Apr 8;10:151. doi: 10.1186/1471-2164-10-151 (PMC2679758; doi:10.1186/1471-2164-10-151)
Supplement: Additional file 3 — Upregulated transcripts in NETKO library. This file contains differentially expressed transcripts that are up-regulated in NETKO cells. [file 1471-2164-10-151-S3.doc]

**Additional file 3: Up-regulated transcripts in NETKO library.** Libraries were normalized as Tag per million (TPM).

| Symbol | Unigene | Description | KO_TPM | WT_TPM | P value | KO/WT |
| --- | --- | --- | --- | --- | --- | --- |
| 2900073G15Rik | Mm.261329 | RIKEN cDNA 1500001M02 gene | 390 | 29 | 0.002 | 13.44827586 |
| Pja2 | Mm.41711 | praja 2, RING-H2 motif containing | 351 | 29 | 0.003 | 12.10344828 |
| Htr3a | Mm.4831 | 5-hydroxytryptamine (serotonin) receptor 3A | 273 | 29 | 0.02 | 9.413793103 |
| Sbk1 | Mm.29660 | SH3-binding kinase | 273 | 0 | 0.003 | 9.413793103 |
| Spc12 | Mm.28852 | RIKEN cDNA 1810004F21 gene | 273 | 29 | 0.02 | 9.413793103 |
| Tlx2 | Mm.37 | T-cell leukemia, homeobox 2 | 273 | 0 | 0.003 | 9.413793103 |
| 2410129E14Rik | Mm.379227 | RIKEN cDNA 2410129E14 gene | 273 | 29 | 0.02 | 9.413793103 |
| Ars2 | Mm.27932 | arsenate resistance protein 2 | 234 | 29 | 0.03 | 8.068965517 |
| C10orf4 | Mm.30607 | RIKEN cDNA 5730455O13 gene | 234 | 29 | 0.03 | 8.068965517 |
| Ysg2 | Mm.196345 | yolk sac gene 2 | 234 | 0 | 0.006 | 8.068965517 |
| DCPSAW986112 | Mm.229110 | RIKEN cDNA 1700001E16 gene | 234 | 0 | 0.006 | 8.068965517 |
| Hoxa10 | Mm.5 | homeo box A10 | 234 | 0 | 0.006 | 8.068965517 |
| 2610507B11Rik | Mm.237103 | RIKEN cDNA 2610507B11 gene | 234 | 0 | 0.006 | 8.068965517 |
| Uhmk1 | Mm.209150 | Uhmk1 U2AF homology motif (UHM) kinase 1 (Uhmk1), mRNA | 234 | 29 | 0.03 | 8.068965517 |
| Map2k2 | Mm.275436 | mitogen activated protein kinase kinase 2 | 234 | 29 | 0.03 | 8.068965517 |
| Pmvk | Mm.34242 | phosphomevalonate kinase | 234 | 29 | 0.03 | 8.068965517 |
| Rela | Mm.249966 | v-rel reticuloendotheliosis viral oncogene homolog A (avian) | 234 | 29 | 0.03 | 8.068965517 |
| Serpinb6a | Mm.252210 | serine (or cysteine) proteinase inhibitor, clade B, member 6a | 234 | 0 | 0.006 | 8.068965517 |
| Shmt2 | Mm.29890 | serine hydroxymethyl transferase 2 (mitochondrial) | 234 | 29 | 0.03 | 8.068965517 |
| Tbrg1 | Mm.28689 | transforming growth factor beta regulated gene 1 | 234 | 0 | 0.006 | 8.068965517 |
| Socs2 | Mm.432401 | suppressor of cytokine signaling 2 | 468 | 58 | 0.002 | 8.068965517 |
| Tnfsf12 | Mm.8983 | tumor necrosis factor (ligand) superfamily, member 13 | 234 | 0 | 0.006 | 8.068965517 |
| Sema4g | Mm.34404 | M.musculus tex271 mRNA (3'region) | 234 | 29 | 0.03 | 8.068965517 |
| Iars | Mm.21118 | Isoleucine-tRNA synthetase | 234 | 29 | 0.03 | 8.068965517 |
| Tfg | Mm.425970 | Trk-fused gene | 234 | 29 | 0.03 | 8.068965517 |
| Agpat1 | Mm.8684 | 1-acylglycerol-3-phosphate O-acyltransferase 1 (lysophosphatidic acid acyltransferase, alpha) | 195 | 0 | 0.02 | 6.724137931 |
| C5orf6 | Mm.29424 | RIKEN cDNA 2810012G03 gene | 195 | 0 | 0.02 | 6.724137931 |
| Ankrd17 | Mm.245522 | ankyrin repeat domain 17 | 195 | 0 | 0.02 | 6.724137931 |
| Glrx | Mm.25844 | glutaredoxin 1 (thioltransferase) | 195 | 0 | 0.02 | 6.724137931 |
| Leprot1 | Mm.34212 | leptin receptor overlapping transcript-like 1 | 195 | 0 | 0.02 | 6.724137931 |
| Cd200 | Mm.245851 | Cd200 antigen (Cd200), mRNA | 195 | 0 | 0.02 | 6.724137931 |
| Ppp2r5d | Mm.295009 | protein phosphatase 2, regulatory subunit B (B56), delta isoform | 390 | 58 | 0.006 | 6.724137931 |
| Xab2 | Mm.23739 | XPA binding protein 2 | 195 | 0 | 0.02 | 6.724137931 |
| Mea1 | Mm.329083 | male enhanced antigen 1 | 390 | 58 | 0.006 | 6.724137931 |
| Cln8 | Mm.254027 | ceroid-lipofuscinosis, neuronal 8 | 195 | 0 | 0.02 | 6.724137931 |
| 2610524G07Rik | Mm.315430 | RIKEN cDNA 2610524G07 gene | 546 | 87 | 0.002 | 6.275862069 |
| Aplp2 | Mm.19133 | amyloid beta (A4) precursor-like protein 2 | 351 | 58 | 0.02 | 6.051724138 |
| D4Wsu53e | Mm.331964 | DNA segment, Chr 4, Wayne State University 53, expressed | 351 | 58 | 0.02 | 6.051724138 |
| Klhl24 | Mm.434398 | Kelch-like 24 (Drosophila) (Klhl24) | 156 | 0 | 0.03 | 5.379310345 |
| Asb6 | Mm.27656 | ankyrin repeat and SOCS box-containing protein 6 | 156 | 0 | 0.03 | 5.379310345 |
| Atp1b1 | Mm.4550 | ATPase, Na+/K+ transporting, beta 1 polypeptide | 156 | 0 | 0.03 | 5.379310345 |
| Islr2 | Mm.186499 | Immunoglobulin superfamily containing leucine-rich repeat 2 (Islr2) | 156 | 0 | 0.03 | 5.379310345 |
| Zmynd11 | Mm.397307 | Zinc finger, MYND domain containing 11 (Zmynd11) | 156 | 0 | 0.03 | 5.379310345 |
| Col9a3 | Mm.141312 | procollagen, type IX, alpha 3 | 156 | 0 | 0.03 | 5.379310345 |
| Cpo | Mm.291519 | coproporphyrinogen oxidase | 156 | 0 | 0.03 | 5.379310345 |
| Cugbp1 | Mm.29495 | CUG triplet repeat, RNA binding protein 1 | 156 | 0 | 0.03 | 5.379310345 |
| Kbtbd2 | Mm.57295 | kelch repeat and BTB (POZ) domain containing 2 | 156 | 0 | 0.03 | 5.379310345 |
| Fbln2 | Mm.249146 | fibulin 2 | 156 | 0 | 0.03 | 5.379310345 |
| Fdps | Mm.39472 | farnesyl diphosphate synthetase | 312 | 58 | 0.03 | 5.379310345 |
| 2610200G18Rik | Mm.46513 | RIKEN cDNA 2610200G18 gene | 156 | 0 | 0.03 | 5.379310345 |
| Gtf2ird2 | Mm.218744 | RIKEN cDNA 1700012P16 gene,GTF2I repeat domain containing 2 | 156 | 0 | 0.03 | 5.379310345 |
| B230219D22Rik | Mm.28449 | RIKEN cDNA B230219D22 gene | 156 | 0 | 0.03 | 5.379310345 |
| Guk1 | Mm.3624 | guanylate kinase 1 | 156 | 0 | 0.03 | 5.379310345 |
| Zcchc11 | Mm.25181 | Zinc finger, CCHC domain containing 11 | 156 | 0 | 0.03 | 5.379310345 |
| Klf13 | Mm.240473 | Kruppel-like factor 13 | 156 | 0 | 0.03 | 5.379310345 |
| Limk1 | Mm.15409 | LIM-domain containing, protein kinase | 156 | 0 | 0.03 | 5.379310345 |
| BC038613 | Mm.59997 | cDNA sequence BC038613 | 156 | 0 | 0.03 | 5.379310345 |
| A530088H08Rik | Mm.209283 | RIKEN cDNA A530088H08 gene | 156 | 0 | 0.03 | 5.379310345 |
| Myo1b | Mm.3390 | myosin IB | 156 | 0 | 0.03 | 5.379310345 |
| Numbl | Mm.255487 | numb-like | 156 | 0 | 0.03 | 5.379310345 |
| Rab3a | Mm.5083 | RAB3A, member RAS oncogene family | 156 | 0 | 0.03 | 5.379310345 |
| Rps16 | Mm.702 | ribosomal protein S16 | 312 | 58 | 0.03 | 5.379310345 |
| Sbds | Mm.280484 | Shwachman-Bodian-Diamond syndrome homolog (human) | 156 | 0 | 0.03 | 5.379310345 |
| Smarca5 | Mm.246803 | RIKEN cDNA 9230115A19 gene, SWI/SNF related, matrix associated, actin dependent regulator of chromatin, subfamily a, member 5 | 156 | 0 | 0.03 | 5.379310345 |
| Sod2 | Mm.290876 | superoxide dismutase 2, mitochondrial | 156 | 0 | 0.03 | 5.379310345 |
| Tubb4 | Mm.7420 | tubulin, beta 4 | 156 | 0 | 0.03 | 5.379310345 |
| Exosc4 | Mm.322752 | Exosome component 4 | 312 | 58 | 0.03 | 5.379310345 |
| Ccnt2 | Mm.268672 | cyclin T2 | 156 | 0 | 0.03 | 5.379310345 |
| Pcbp4 | Mm.286394 | poly(rC) binding protein 4 | 156 | 0 | 0.03 | 5.379310345 |
| Mier2 | Mm.334193 | Mesoderm induction early response 1, family member 2 | 156 | 0 | 0.03 | 5.379310345 |
| Sec24d | Mm.260039 | SEC24 related gene family, member D | 156 | 0 | 0.03 | 5.379310345 |
| Smarca5 | Mm.246803 | SWI/SNF related, matrix associated, actin dependent regulator of chromatin, subfamily a, member 5 | 156 | 0 | 0.03 | 5.379310345 |
| 1110001P04Rik | Mm.28869 | Mus musculus 18-day embryo whole body cDNA, RIKEN full-length enriched library, clone:1110001P04 product:unknown EST, full insert sequence | 273 | 58 | 0.05 | 4.706896552 |
| Cpsf5 | Mm.28961 | cleavage and polyadenylation specific factor 5 | 273 | 58 | 0.05 | 4.706896552 |
| Cript | Mm.109329 | Cysteine-rich PDZ-binding protein | 273 | 58 | 0.05 | 4.706896552 |
| Limd2 | Mm.21687 | LIM domain containing 2 | 273 | 58 | 0.05 | 4.706896552 |
| Ret | Mm.57199 | ret proto-oncogene | 273 | 58 | 0.05 | 4.706896552 |
| Th | Mm.1292 | tyrosine hydroxylase | 273 | 58 | 0.05 | 4.7 |
| Dpp3 | Mm.234769 | dipeptidylpeptidase 3 | 390 | 87 | 0.02 | 4.482758621 |
| Ldhb | Mm.9745 | lactate dehydrogenase 2, B chain | 390 | 87 | 0.02 | 4.482758621 |
| Dbh | Mm.167781 | dopamine beta hydroxylase | 351 | 87 | 0.03 | 4.034482759 |
| Mtap1b | Mm.4173 | Microtubule-associated protein 1 B | 351 | 87 | 0.03 | 4.034482759 |
| Chgb | Mm.255241 | chromogranin B | 975 | 261 | 0.0001 | 3.735632184 |
| Nnat | Mm.233903 | neuronatin | 624 | 174 | 0.006 | 3.586206897 |
| Uchl1 | Mm.29807 | ubiquitin carboxy-terminal hydrolase L1 | 1248 | 348 | 0.0001 | 3.586206897 |
| Adam10 | Mm.3037 | a disintegrin and metalloprotease domain 10 | 507 | 145 | 0.02 | 3.496551724 |
| 2310044H10Rik | Mm.294759 | RIKEN cDNA 2310044H10 gene | 507 | 145 | 0.02 | 3.496551724 |
| Klhdc2 | Mm.234368 | kelch domain containing 2 | 468 | 145 | 0.03 | 3.227586207 |
| Ppib | Mm.390199 | peptidylprolyl isomerase B | 468 | 145 | 0.03 | 3.227586207 |
| Litaf | Mm.294753 | LPS-induced TN factor | 741 | 232 | 0.005 | 3.193965517 |
| 1500032D16Rik | Mm.28349 | RIKEN cDNA 1500032D16 gene | 429 | 145 | 0.05 | 2.95862069 |
| Tgfb2 | Mm.18213 | transforming growth factor, beta 2 | 429 | 145 | 0.05 | 2.95862069 |
| Txnl4 | Mm.172411 | Thioredoxin-like 4 | 507 | 174 | 0.03 | 2.913793103 |
| S100a6 | Mm.100144 | S100 calcium binding protein A6 (calcyclin) | 507 | 174 | 0.03 | 2.913793103 |
| Tde2 | Mm.29344 | tumor differentially expressed 2 | 507 | 174 | 0.03 | 2.913793103 |
| Scoc | Mm.246911 | short coiled-coil protein | 585 | 203 | 0.02 | 2.881773399 |
| Nef3 | Mm.242832 | neurofilament 3, medium | 1014 | 377 | 0.004 | 2.689655172 |
| Prph | Mm.2477 | peripherin 1 | 1092 | 406 | 0.003 | 2.689655172 |
| Tmem59 | Mm.291192 | Transmembrane protein 59 | 468 | 174 | 0.05 | 2.689655172 |
| Mll2 | Mm.425955 | Myeloid/lymphoid or mixed-lineage leukemia 2 | 858 | 319 | 0.007 | 2.689655172 |
| Enol | Mm.70666 | enolase 1, alpha non-neuron | 3861 | 1450 | 0.001 | 2.662758621 |
| Mgp | Mm.243085 | matrix gamma-carboxyglutamate (gla) protein | 975 | 377 | 0.006 | 2.586206897 |
| Tubb3 | Mm.40068 | tubulin, beta 3 | 819 | 319 | 0.02 | 2.567398119 |
| Pdia3 | Mm.263177 | Grp58 Protein disulfide isomerase associated 3, mRNA | 663 | 261 | 0.03 | 2.540229885 |
| App | Mm.277585 | amyloid beta (A4) precursor protein | 1248 | 551 | 0.005 | 2.264972777 |
| Ldha | Mm.29324 | lactate dehydrogenase 1, A chain | 2145 | 957 | 0.001 | 2.24137931 |
| Igfbp5 | Mm.405761 | insulin-like growth factor binding protein 5 | 741 | 348 | 0.05 | 2.129310345 |
| Hnrpk | Mm.142872 | heterogeneous nuclear ribonucleoprotein K | 897 | 435 | 0.04 | 2.062068966 |
| GTL2 | Mm.289645 | GTL2, imprinted maternally expressed untranslated mRNA | 1092 | 551 | 0.03 | 1.98185118 |
| Cartpt | Mm.75498 | CART prepropeptide (Cartpt) | 429 | 232 | 0.05 | 1.849137931 |
| GNAS | Mm.125770 | GNAS (guanine nucleotide binding protein, alpha stimulating) complex locus | 2613 | 1537 | 0.004 | 1.700065062 |
| Eef1b2 | Mm.2718 | eukaryotic translation elongation factor 1 beta 2 | 1716 | 1044 | 0.04 | 1.643678161 |
| Fau | Mm.391633 | Finkel-Biskis-Reilly murine sarcoma virus (FBR-MuSV) ubiquitously expressed (fox derived) | 3003 | 1885 | 0.008 | 1.593103448 |

The ratio value (NET KO/WT): the occurrence in NET KO library compared to the WT library, to avoid division by zero, we have assumed one tag if no tag was detected. The P Value were calculated according to this paper: <http://www.genome.org/cgi/content/full/7/10/986> , and in website: <http://igs-server.cnrs-mrs.fr/~audic/winflat.cgi> .
