# Supplementary material for: Norepinephrine transport-mediated gene expression in noradrenergic neurogenesis
Source: BMC Genomics. 2009 Apr 8;10:151. doi: 10.1186/1471-2164-10-151 (PMC2679758; doi:10.1186/1471-2164-10-151)
Supplement: Additional file 4 — Downregulated transcripts in NETKO library. This file contains differentially expressed transcripts that are down-regulated in NETKO cells. [file 1471-2164-10-151-S4.doc]

**Additional File 4: Down-regulated transcripts in NETKO library.** Libraries were normalized as Tag per million (TPM).

| Symbol | Unigene | Description | KO_TPM | WT_TPM | P value | WT/KO |
| --- | --- | --- | --- | --- | --- | --- |
| Cand2 | Mm.277683 | cullin-associated and neddylation-dissociated 2 (putative) | 39 | 580 | 0.001 | 14.87179487 |
| Nmnat1 | Mm.76062 | nicotinamide nucleotide adenylyl-transferase 1 | 39 | 551 | 0.001 | 14.12820513 |
| Palld | Mm.29933 | palladin, cytoskeletal associated protein | 39 | 435 | 0.002 | 11.15384615 |
| Sec24d | Mm.29293 | RIKEN cDNA 2310020L09 gene, SEC24 related gene family, member D | 39 | 377 | 0.006 | 9.666666667 |
| Serf1 | Mm.286177 | small EDRK-rich factor 1 | 0 | 348 | 0.002 | 8.923076923 |
| Kctd12 | Mm.246466 | potassium channel tetramerisation domain containing 12 | 0 | 319 | 0.003 | 8.179487179 |
| Khdrbs1 | Mm.8256 | KH domain containing, RNA binding, signal transduction associated 1 | 39 | 290 | 0.03 | 7.435897436 |
| Myh9 | Mm.29677 | myosin heavy chain IX | 39 | 290 | 0.03 | 7.435897436 |
| Rpl32 | Mm.104368 | ribosomal protein L32 | 78 | 580 | 0.001 | 7.435897436 |
| 3321401G04Rik | Mm.24652 | RIKEN cDNA 3321401G04 gene | 39 | 261 | 0.04 | 6.692307692 |
| 4933440N22Rik | Mm.122366 | RIKEN cDNA 4833420I20 gene | 39 | 261 | 0.04 | 6.692307692 |
| Akt1 | Mm.6645 | thymoma viral proto-oncogene 1 | 39 | 261 | 0.04 | 6.692307692 |
| Tshz1 | Mm.102136 | teashirt zinc finger family member 1, serologically defined colon cancer antigen 33, | 39 | 261 | 0.04 | 6.692307692 |
| G3bp | Mm.39631 | Ras-GTPase-activating protein SH3-domain binding protein | 39 | 261 | 0.04 | 6.692307692 |
| Hip1 | Mm.280805 | huntingtin interacting protein 1 | 0 | 261 | 0.008 | 6.692307692 |
| Sdbh | Mm.246965 | succinate dehydrogenase complex, subunit B, iron sulfur (Ip) | 39 | 261 | 0.04 | 6.692307692 |
| Rnaset2 | Mm.181237 | ribonuclease T2 | 39 | 261 | 0.04 | 6.692307692 |
| Rab18 | Mm.132802 | RAB18, member RAS oncogene family | 0 | 232 | 0.02 | 5.948717949 |
| Bmp6 | Mm.385759 | bone morphogenetic protein 6 | 0 | 232 | 0.02 | 5.948717949 |
| Polr2i | Mm.423030 | polymerase (RNA) II (DNA directed) polypeptide I | 0 | 232 | 0.02 | 5.948717949 |
| Hdac2 | Mm.19806 | histone deacetylase 2 | 39 | 232 | 0.05 | 5.9487179 |
| Ddx39 | Mm.28222 | DEAD (Asp-Glu-Ala-Asp) box polypeptide 39 | 39 | 232 | 0.05 | 5.9487179 |
| Bcas2 | Mm.104919 | breast carcinoma amplified sequence 2 | 0 | 203 | 0.03 | 5.205128205 |
| Cdc5l | Mm.28270 | cell division cycle 5-like (S. pombe) | 0 | 203 | 0.03 | 5.205128205 |
| Col11a1 | Mm.209715 | procollagen, type XI, alpha 1 | 0 | 203 | 0.03 | 5.205128205 |
| Ctbp1 | Mm.7286 | C-terminal binding protein 1 | 0 | 203 | 0.03 | 5.205128205 |
| Epm2aip1 | Mm.209005 | EPM2A (laforin) interacting protein 1 | 0 | 203 | 0.03 | 5.205128205 |
| Hoxb9 | Mm.258271 | homeo box B9 | 0 | 203 | 0.03 | 5.205128205 |
| Iqgap1 | Mm.207619 | IQ motif containing GTPase activating protein 1 | 0 | 203 | 0.03 | 5.205128205 |
| Mcm7 | Mm.241714 | Mus musculus adult male thymus cDNA, RIKEN full-length enriched library, clone:5830410A10 product:mini chromosome maintenance deficient 7 (S. cerevisiae), full insert sequence | 0 | 203 | 0.03 | 5.205128205 |
| Txndc5 | Mm.28622 | thioredoxin domain containing 5 | 0 | 203 | 0.03 | 5.205128205 |
| Mdc1 | Mm.218511 | Mediator of DNA damage checkpoint 1 | 0 | 203 | 0.03 | 5.205128205 |
| Pea15 | Mm.544 | phosphoprotein enriched in astrocytes 15 | 0 | 203 | 0.03 | 5.205128205 |
| Sgta | Mm.30068 | small glutamine-rich tetratricopeptide repeat (TPR)-containing, alpha | 0 | 203 | 0.03 | 5.205128205 |
| RP23-336J1.4 | [Mm.39631](http://www.ncbi.nlm.nih.gov/projects/SAGE/index.cgi?cmd=ugsearch&org=Mm&ug=39631&anchor=NLAIII) | G3bp Ras-GTPase-activating protein SH3-domain binding protein, mRNA (cDNA clone MGC:13925 IMAGE:4020362) | 0 | 203 | 0.03 | 5.205128205 |
| Ppp1r12a | Mm.422959 | protein phosphatase 1, regulatory (inhibitor) subunit 12A | 0 | 203 | 0.03 | 5.205128205 |
| Rhoa | Mm.757 | ras homolog gene family, member A | 78 | 377 | 0.02 | 4.833333333 |
| Matr3 | Mm.215034 | matrin 3 | 78 | 348 | 0.04 | 4.461538462 |
| Aars | Mm.24174 | alanyl-tRNA synthetase | 0 | 174 | 0.04 | 4.461538462 |
| Actg2 | Mm.292865 | actin, gamma 2, smooth muscle, enteric | 0 | 174 | 0.04 | 4.461538462 |
| Ranbp5 | Mm.221452 | RAN binding protein 5 | 0 | 174 | 0.04 | 4.461538462 |
| Bcar1 | Mm.3758 | breast cancer anti-estrogen resistance 1 | 0 | 174 | 0.04 | 4.461538462 |
| Erbb3 | Mm.29023 | v-erb-b2 erythroblastic leukemia viral oncogene homolog 3 (avian) | 0 | 174 | 0.04 | 4.461538462 |
| Hmgn1 | Mm.2756 | high mobility group nucleosomal binding domain 1 | 0 | 174 | 0.04 | 4.461538462 |
| Ifi30 | Mm.30241 | interferon gamma inducible protein 30 | 0 | 174 | 0.04 | 4.461538462 |
| Arl6ip6 | Mm.37623 | ADP-ribosylation factor-like 6 interacting protein 6 | 0 | 174 | 0.04 | 4.461538462 |
| Nola3 | Mm.272346 | nucleolar protein family A, member 3 | 0 | 174 | 0.04 | 4.461538462 |
| Ranbp2 | Mm.142730 | RAN binding protein 2 | 0 | 174 | 0.04 | 4.461538462 |
| Retnla | Mm.33772 | resistin like alpha | 0 | 174 | 0.04 | 4.461538462 |
| 4921524J06Rik | Mm.35296 | RIKEN cDNA 4921524J06 gene | 0 | 174 | 0.04 | 4.461538462 |
| Tsll2 | Mm.178322 | TSLC1-like 2 | 0 | 174 | 0.04 | 4.461538462 |
| Epc1 | Mm.312133 | enhancer of polycomb homolog 1 (Drosophila) | 0 | 174 | 0.04 | 4.461538462 |
| Eif3s10 | Mm.2238 | eukaryotic translation initiation factor 3, subunit 10 (theta) | 0 | 174 | 0.04 | 4.461538462 |
| Mars | Mm.28173 | methionine-tRNA synthetase | 0 | 174 | 0.04 | 4.461538462 |
| Bola2 | [Mm.358692](http://www.ncbi.nlm.nih.gov/projects/SAGE/index.cgi?cmd=ugsearch&org=Mm&ug=358692&anchor=NLAIII) | 1110025L05Rik BolA-like 2 (E. coli), mRNA (cDNA clone MGC:74338 IMAGE:6707992) | 0 | 174 | 0.04 | 4.461538462 |
| Nexn | [Mm.200188](http://www.ncbi.nlm.nih.gov/projects/SAGE/index.cgi?cmd=ugsearch&org=Mm&ug=200188&anchor=NLAIII) | Nexn Nexilin (Nexn), mRNA | 0 | 174 | 0.04 | 4.461538462 |
| Ube2j1 | Mm.259095 | ubiquitin-conjugating enzyme E2, J1 | 0 | 174 | 0.04 | 4.461538462 |
| Zfp36l1 | Mm.235132 | zinc finger protein 36, C3H type-like 1 | 0 | 174 | 0.04 | 4.461538462 |
| Hmgn2 | Mm.319660 | Hmgn2 High mobility group nucleosomal binding domain 2 (Hmgn2), mRNA | 117 | 493 | 0.02 | 4.213675214 |
| Paics | Mm.182931 | phosphoribosylaminoimidazole carboxylase, phosphoribosylaminoribosylaminoimidazole, succinocarboxamide synthetase | 78 | 319 | 0.05 | 4.08974359 |
| Cox6b1 | Mm.400 | cytochrome c oxidase, subunit VIb | 195 | 725 | 0.003 | 3.717948718 |
| Hspa8 | Mm.336743 | heat shock protein 8 | 390 | 1015 | 0.005 | 2.602564103 |
| Hmgn2 | Mm.911 | high mobility group nucleosomal binding domain 2 | 273 | 638 | 0.05 | 2.336996337 |
| Ddx3x | Mm.289662 | DEAD/H (Asp-Glu-Ala-Asp/His) box polypeptide 3, X-linked | 390 | 870 | 0.03 | 2.230769231 |
| Col1a1 | Mm.277735 | procollagen, type I, alpha 1 | 546 | 1102 | 0.02 | 2.018315018 |
| Ptma | Mm.19187 | prothymosin alpha | 1092 | 2204 | 0.001 | 2.018315018 |
| Hspa8 | Mm.290774 | heat shock protein 8 | 624 | 1189 | 0.03 | 1.905448718 |

The ratio value (NET KO/WT): the occurrence in NET KO library compared to the WT library, to avoid division by zero, we have assumed one tag if no tag was detected. The P Value were calculated according to this paper: <http://www.genome.org/cgi/content/full/7/10/986> , and in website: <http://igs-server.cnrs-mrs.fr/~audic/winflat.cgi> .
