# Supplementary material for: Norepinephrine transport-mediated gene expression in noradrenergic neurogenesis
Source: BMC Genomics. 2009 Apr 8;10:151. doi: 10.1186/1471-2164-10-151 (PMC2679758; doi:10.1186/1471-2164-10-151)
Supplement: Additional file 5 — List of primers used. These two tables list the primers used. [file 1471-2164-10-151-S5.doc]

**ADDITIONAL FILE 5**. List of primers used

| Gene | Up | Lower | Product length |
| --- | --- | --- | --- |
| TH | AGCCGTCTCAGAGCAGGATACCAA | TCAGACACCCGACGCACAGAACT | 414 bp |
| DBH | CTTCCCCCGACACCACATCATCA | CCGGTCAACAAAGGCAGTCTCC | 444 bp |
| MAOa | TTGCCCGGAAAGCTGAACGAC | TCTCTGTGCCTGCAAAGTAAATCC | 249 bp |
| COMT | CTGGGGGTTGGTGGCTATTGGT | GTGCCCCGATGAGGATGGAAACT | 416 bp |
| CART | GCGCCGCCCTGCTGCTACT | GATCCTGGCCCCTTTCCTCACTG | 290 bp |
| HPRT | CCTGCTGGATTACATTAAAGCACTG | CCTGAAGTACTCATTATAGTCAAGG | 370 bp |
| 5-HT3a | TGGCGATCACCGGAAGAAGT | CAGCCGCACAATGAAGATGG | 420 bp |
| NET | CCATACCAAATACTCCAAATACAAG | CGTGAAGAGTTTCCGGTGTCGCTT | 745bp |

Primers purchased from SuperArray

| Gene symbol | Cat # | description |
| --- | --- | --- |
| Numbl | PPM41828E | Numb-like |
| Pja2 | PPM28882A | Praja 2, RING-H2 motif containing |
| Hdac2 | PPM04361E | Histone deacetylase 2 |
| Tlx2 | PPM24482A | T-cell leukemia, homeobox 2 |
